# Antibacterial Electrospun Membrane with Hierarchical Bead-on-String Structured Fibres for Wound Infections

**DOI:** 10.3390/nano14171429

**Published:** 2024-08-31

**Authors:** Yu Xuan Fong, Catherine Pakrath, Fathima Shana Pattar Kadavan, Tien Thanh Nguyen, Trong Quan Luu, Borislav Stoilov, Richard Bright, Manh Tuong Nguyen, Neethu Ninan, Youhong Tang, Krasimir Vasilev, Vi Khanh Truong

**Affiliations:** 1 College of Medicine and Public Health, Flinders University, Bedford Park, Adelaide, SA 5042, Australia; 2 Institute for NanoScale Science and Technology, College of Science and Engineering, Flinders University, Bedford Park, Adelaide, SA 5042, Australia; 3 Medical Device Research Institute, College of Science and Engineering, Flinders University, Bedford Park, Adelaide, SA 5042, Australia

**Keywords:** nanofibres, polycaprolactone, silk sericin, biocompatible, biodegradable, natural polymer, ciprofloxacin

## Abstract

Chronic wounds often result in multiple infections with various kinds of bacteria and uncontrolled wound exudate, resulting in several healthcare issues. Advanced medicated nanofibres prepared by electrospinning have gained much attention for their topical application on infected chronic wounds. The objective of this work is to enhance the critical variables of ciprofloxacin-loaded polycaprolactone-silk sericin (PCL/SS-PVA-CIP) nanofibre production via the process of electrospinning. To examine the antibacterial effectiveness of PCL/SS-PVA-CIP nanocomposites, the material was tested against *P. aeruginosa* and *S. aureus*. The combination of PCL/SS-PVA-CIP exhibited potent inhibitory properties, with the most effective concentrations of ciprofloxacin (CIP) being 3 μg/g and 7.0 μg/g for each bacterium, respectively. The biocompatibility was evaluated by conducting cell reduction and proliferation studies using the human epidermal keratinocyte (HaCaT) cells and human gingival fibroblasts (HGFs) in vitro cell lines. The PCL/SS-PVA-CIP showed good cell compatibility with HaCaT and HGF cells, with effective proliferation even at antibiotic doses of up to 7.0 μg/g. The drug release effectiveness of the nanocomposites was assessed at various concentrations of CIP, resulting in a maximum cumulative release of 76.5% and 74.4% after 72 h for CIP concentrations of 3 μg/g and 7 μg/g, respectively. In summary, our study emphasizes the possibility of combining silk sericin (SS) and polycaprolactone (PCL) loading with CIP nanocomposite for wound management.

## 1. Introduction

Bacterial infections pose a persistent global public health challenge, causing significant morbidity and mortality [1,2,3]. They slow the physiological wound-healing process. Pathogenic bacteria secrete various substances that prolong the inflammatory phase of wound healing after colonisation, thereby delaying the wound healing cascade [4,5,6]. Acute wounds are generally not infected and heal without the need for antibiotics. Infected chronic wounds, on the other hand, require local antibiotics for effective wound management [7,8,9]. Although antibiotics have revolutionized wound management, their efficacy is increasingly undermined by the emergence of antibiotic-resistant strains [10]. The selective pressure exerted by antibiotics encourages the survival of resistant bacteria, and the cytotoxicity of some antibiotics towards mammalian cells can limit their use due to undesirable side effects [11]. Additionally, the lack of precise targeting by current antibiotic delivery systems can lead to systemic exposure, harming even healthy tissues [9].

Addressing these challenges require innovative strategies for combating bacterial infections. Nanofibrous scaffolds are emerging as a versatile and effective platform for targeted antibiotic delivery [12]. Their ability to directly transport drugs to the site of infection in a controlled manner offers sustained therapeutic benefits [13]. They can reduce the risk of developing antibiotic resistance by minimizing exposure of bacteria to sub-lethal antibiotic concentrations [14,15]. Electrospinning is a top-down approach for fabricating nanofibrous scaffolds with diameters varying from nanometers to micrometers [16,17]. Their high surface area, three-dimensional reticular structures, and high porosity make them ideal candidates for various biomedical applications. However, one of the significant challenges in electrospinning for medical applications is achieving consistent reproducibility and scalability of the process. Industrial scale-up remains complex due to variables such as polymer solution properties, ambient conditions, and control over fiber deposition [18]. Conventional, co-axial, and emulsion electrospinning techniques are employed to synthesize drug-loaded nanofibres [19,20]. In conventional electrospinning, drugs are directly integrated into nanofibres either by electrospinning polymer/drug solution or adsorption of drugs after electrospinning [21,22]. Among the challenges in the field are the difficulties in controlling the release kinetics of loaded bioactive agents, which can result in an undesirable burst release, which can lead to toxicity [23,24], instead of the preferred sustained release. Additionally, the mechanical properties of electrospun fibres often fall short of the robustness required for certain types of wound dressings, particularly those designed for load-bearing areas [25]. Coaxial and emulsion electrospinning can minimize the burst release of drugs by encapsulating them in the nanofibres and avoiding the use of organic solvents [26,27]. Recent advancements have focused on integrating bioactive molecules, such as growth factors, antibiotics, and anti-inflammatory agents, directly into the fibres. Innovations like dual-drug delivery systems and the use of conductive polymers have demonstrated enhanced wound healing by providing sustained therapeutic release and supporting cellular activities that promote tissue regeneration [25]. The polymeric outer shell will act as a barrier facilitating the sustained release of the drug. Bead-on-string fibres generated through emulsion electrospinning have several advantages in drug delivery. The presence of beads in nanofibrous scaffolds can control the surface area, pore size distribution, and mechanical properties, and offers opportunities for multi-functional usages [28,29,30]. Herein, we have explored the application of emulsion electrospinning to synthesize drug-loaded bead-on-string composite nanofibrous scaffolds.

Both synthetic and natural polymers are used for synthesizing nanofibrous electrospun scaffolds [31,32]. Despite their potential, the use of synthetic polymers in the fabrication of such nanofibres frequently encounters limitations related to bioactivities, such as poor cell adhesion, migration, proliferation, and differentiation [33,34]. These shortcomings can significantly slow the process of tissue regeneration. Hence, it requires significant modification for biomedical use [35]. A promising solution is the integration of synthetic polymers with natural materials. Silk sericin (SS) [36], a natural macromolecule, is renowned for its antioxidant, antibacterial, biocompatible, and hydrophilic properties, making it ideal for moisture-absorbing applications [37,38,39,40]. Incorporating SS into nanofibres, such as those made from poly(ε-caprolactone) (PCL) and polyvinyl alcohol (PVA), can improve mechanical strength, degradation rates, and biocompatibility [41,42,43]. Additionally, SS can enhance cellular adhesion, proliferation, and extracellular matrix production, benefiting tissue engineering applications [44,45].

This study introduces a facile approach to fabricate a bead-on-string SS-based nanofibrous composite scaffold created via electrospinning, designed for localized antibiotic delivery. Ciprofloxacin (CIP) is the model drug employed for this study due to its broad antibiotic spectrum [34]. By combining ciprofloxacin-loaded PCL and SS with a PVA coating, we produced PCL/SS-PVA-CIP nanofibres. We detail the fabrication, characterization, and evaluation of these scaffolds, focusing on their potential bacterial infection management while mitigating the risks associated with antibiotic resistance and toxicity.

## 2. Materials and Methods

### 2.1. Materials

The electrospinning polymer emulsion was comprised of silk sericin powder (99.9% purity) obtained from Nano Silk Medtech, Ho Chi Minh, Vietnam, and polycaprolactone (PCL) (Mn 45,000), poly(vinyl alcohol) (Mn 85,000–124,000), and Tween-20 (10% *w*/*v*) sourced from Sigma-Aldrich, Steinheim, Germany. During the studies, beakers and vials were washed using ultra-pure Milli Q water (Millipore Milli-Q Academic, France) with a resistivity of more than 18 MΩ·cm. Glacial acetic acid (0.5% *w*/*v*), ethyl acetate, phosphate-buffered saline (PBS) tablets, MTT reagent and dimethyl sulfoxide (DMSO) were purchased from Sigma Aldrich, Steinheim, Germany. Dulbecco’s Modified Eagle’s Medium (DMEM/F12), Foetal Bovine Serum (FBS), and Penicillin–Streptomycin were obtained from Thermo Fisher, New York, NY, USA. Tryptone Soy Broth (TSB) was procured from Oxoid, Basingstoke, UK.

### 2.2. Methods

#### 2.2.1. Nanofibre Fabrication

**Scheme 1:**
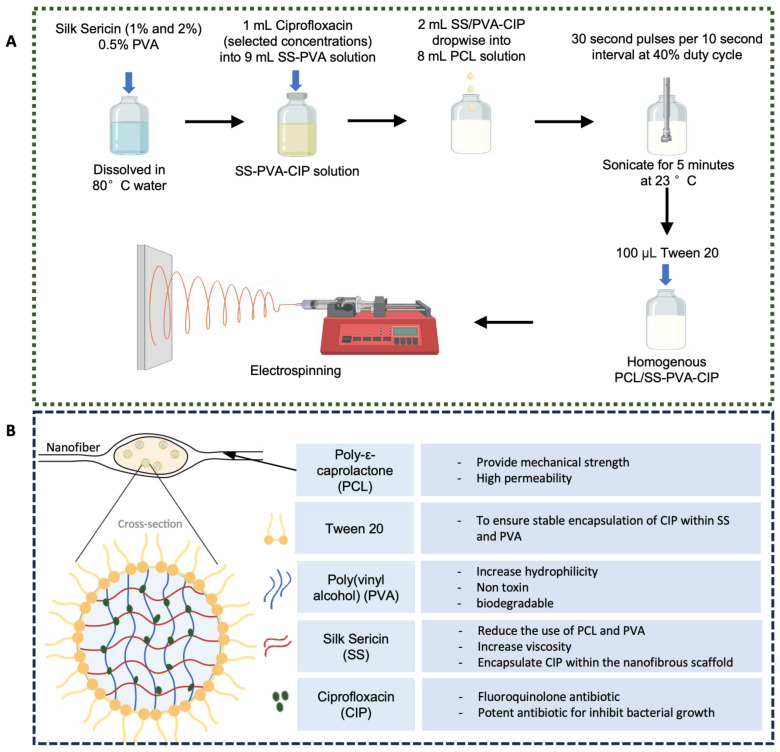
(**A**) Schematic of the PCL/SS-PVA-CIP nanofibre fabrication process. (**B**) The cross-sectional area of the encapsulation of CIP within SS and PVA with Tween 20.

The PCL solution was made by dissolving 10 wt% PCL in ethyl acetate on a magnetic stirrer at 1200 rpm for three hours. PVA was used to encapsulate the silk sericin by dissolving both polymers in the same aqueous phase. To generate the drug-loaded SS-PVA solution, 0.5 wt% PVA was dissolved in Milli-Q water at 80 °C on a magnetic stirrer operating at 800 rpm with 1% or 2% SS. After the SS and PVA had completely dissolved, the solution was allowed to cool down at room temperature. CIP was added to the SS-PVA solutions at concentrations of 70 μg/mL, 140 μg/mL, and 320 μg/mL, using a polymer-solution-to-drug ratio of 9:1. The final concentrations of CIP were 1.4 μg/mL, 2.8 μg/mL, and 6.4 μg/mL respectively. The electrospinning emulsion was formulated by gradually introducing the SS-PVA-CIP solution into the PCL solution at a ratio of 1:4, while maintaining continuous agitation at 700 rpm for 30 min. The electrospinning emulsion underwent sonication for 5 min, with a pulse time of 30 s for every 10 s interval. Afterwards, to achieve greater uniformity in the PCL/SS-PVA-CIP composition, 100 μL of Tween 20 was gradually added to the emulsion, with slow stirring continuously for 10 min. The final emulsion was subjected to sonication for an additional 5 min using identical parameters and subsequently allowed to cool. Scheme 1A illustrates how electrospinning was utilized to fabricate nanofibrous scaffolds from the prepared emulsion. Prior to the construction of the fibres, the collector was enveloped in aluminium foil. Table 1 shows the final concentration of CIP in the nanofibre scaffolds.

#### 2.2.2. Viscosity and Conductivity of Polymer Emulsion

The shear viscosities or dynamic viscosities of solutions containing 10% PCL, SS-PVA-CIP, and PCL/SS-PVA-CIP were determined using an AR 2000 Rheometer (TA Instruments, UK). The rheometer was adjusted to a temperature of 23 °C to correspond with the temperature of the electrospinning chamber. The samples were first cast at the pre-heated Peltier plate, and a 25 mm aluminium parallel plate geometry was used, at a 100 μm gap distance to perform the study. The electrical conductivity of emulsions was assessed by employing a multimeter under ambient conditions. A thermocouple thermometer was used to measure the temperature within the chamber during the electrospinning operation.

#### 2.2.3. Morphological Structures

The morphological structures of fabricated nanofibres were examined using a scanning electron microscope (FEI Inspect F50 Field Emission SEM, FEI Company, USA). To minimize the occurrence of charge artefacts and improve the visibility of the nanofibres in the SEM images, the samples were coated with 5 nm platinum layers using an Emitech K75X single-target sputter coater, Germany. The diameter of the nanofibres was measured by analyzing the SEM images with ImageJ software (version 1.54g 18 October 2023). Fifty nanofibres were selected at random from the photographs, and their mean diameters were measured.

#### 2.2.4. Wettability Analysis

The wettability of the produced nanofibres was assessed by measuring the contact angle formed by water droplets at the boundary between the liquid and solid surfaces [46]. To obtain uniform and reproducible measurements, all samples were prepared with the same dimensions of 10 mm × 10 mm. Prior to conducting contact angle measurements, the samples underwent a meticulous drying process and were smoothed to minimize any surface defects. Each sample was deposited with a 5 μL sessile droplet of water using a syringe. Afterwards, advanced high-resolution digital microscopy was used to acquire photographs of the water droplets on the surfaces of the scaffold. The contact angles of three repeated measurements for each scaffold composition were obtained using image analysis tools, namely the drop-snake tool in ImageJ software (version 1.54g 18 October 2023). The contact angles were measured, averaged, and recorded.

#### 2.2.5. Mass Loss Study

To evaluate the in vitro degradation of the electrospun nanocomposite scaffolds, samples were prepared with uniform dimensions of 10 mm × 10 mm, and their initial weights (*w_i_*) were precisely measured using a digital balance with a sensitivity of 10^−4^ g. Subsequently, these samples were submerged in a PBS solution at pH 7.4 and incubated at 37 °C. At predetermined time points of 1, 3, and 7 days, the scaffolds were carefully removed from the PBS, gently placed on filter paper to remove excess fluid, rinsed with distilled water, and then dried in a vacuum oven for 30 min. After drying, the samples were re-weighed on a digital balance to ascertain their final weights (*w_f_*). The percentage of weight loss, indicative of scaffold degradation, was calculated using the following equation:

(1) $$Degradation\;of\;Scaffold\left(\%\right)=\frac{wi-wf}{wi}\times 100\;$$

where *w_f_* represents the final weight of the scaffold and *w_i_* is the initial weight. This methodology allows for a quantitative assessment of the degradation rate of the nanocomposite scaffolds over time.

#### 2.2.6. Antibacterial Assays

The qualitative analysis were been done using samples with consistent radius of 3 mm. Filter sheets with the same diameters were used as negative controls to determine any potential restrictions on bacterial growth under the specified conditions. Prior to the bacterial incubation, all of the samples were sterilized using uv light. The bacterial strains used for the zone of inhibition [34] assay were the Gram-negative bacterium *Pseudomonas aeruginosa* ATCC 15692 and the Gram-positive bacterium *Staphylococcus aureus* ATCC 25923. The strains were incubated overnight in Tryptic Soy Broth (TSB) stock solutions, using initial inoculums collected from single colonies. Afterwards, the bacterial stock solutions were analyzed for optical density using a BMG Labtech SPECTROstar Nano microplate reader, measuring the absorbance at 600 nm. A reading of 1 at a wavelength of 600 nm equated to roughly 1 × 10^9^ colony-forming units per millilitre (CFU/mL). The stock solutions were diluted with PBS based on the OD values to generate bacterium broths with a bacterial concentration of 1 × 10^8^ CFU/mL for the ZOI assay. Subsequently, 100 μL portions of the bacterial suspensions were equally distributed onto nutritional agar plates in standard 100 mm Petri dishes. Following inoculation, the dishes were dried in a biosafety cabinet under sterile conditions. Subsequently, three identical sets of the aseptic scaffold discs were carefully placed into the solidified cultures and placed under incubation at a temperature of 37 °C for 24 h. After the incubation period, any observable areas of inhibition surrounding the discs were quantified and documented as proof of the scaffold’s antimicrobial characteristics.

In addition, we conducted MIC experiments to determine the minimum concentration required to inhibit microbial growth. The concentrations of CIP used in the experiment varied between 100 and 800 μg/mL and between 120 and 250 μg/mL, respectively, for *P. aeruginosa* and *S. aureus*.

#### 2.2.7. Cell Culture

The HaCaT cell line, derived from adult human skin keratinocytes (300493), was acquired from cell line services in Eppelheim, Germany. The HGF cell line, consisting of human gingival fibroblasts (CRL-4061 hTERT), was obtained from ATCC and cultured in Dulbecco’s Modified Eagle Medium (DMEM) supplemented with 10% foetal bovine serum (FBS) and 1% penicillin/streptomycin (Gibco, Thermo Fisher Scientific). The cell lines were cultured at a temperature of 37 °C and in an atmosphere containing 5% carbon dioxide. The media was replaced every 2–3 days, and both cell lines were transferred to other culture dishes when they reached 80% confluency.

#### 2.2.8. Cell Viability and Proliferation

To evaluate the compatibility of the nanofibrous scaffold with cells, HaCaT and HGF cells were cultured in full medium (with 10% FBS) at densities of 10^5^ and 5 × 10^5^ cells per well in 96-well plates, respectively. The assessment focused on cell viability and proliferation. Cells were exposed to a sterilized scaffold test sample 24 h after seeding, under the conditions of 37 °C and 5% CO_2_, using either full medium or serum-free conditions. The cells lacking a scaffold were exposed to a concentration of 0.5 mg/mL MTT at a temperature of 37 °C and 5% CO_2_ for 2 h. Subsequently, the formazan crystal was dissolved in DMSO for 10 min. Measurement of absorbance was conducted at a wavelength of 570 nm.

#### 2.2.9. Statistical Analysis

Data are shown as mean ± SD. Statistical analysis between groups was performed using one-way ANOVA and the Tukey test using GraphPad Prism 9 (GraphPad Software, San Diego, CA, USA), with significance set at *p* ≤ 0.005.

## 3. Results

### 3.1. Optimal Parameters for Electrospinning

The fabrication of the PCL composite nanofibres involved the strategic incorporation of CIP into SS-PVA solution, subsequently blended into a PCL solution stabilized by Tween 20 (Scheme 1). The introduction of a hydrophilic material like silk sericin (SS) into the PCL matrix enhances the overall hydrophilicity of the composite material. This increased hydrophilicity facilitates greater water absorption, which in turn accelerates the hydrolytic degradation of the ester bonds within PCL. This effect has been demonstrated in our water contact angle studies, where the incorporation of SS significantly reduced the contact angle, indicating improved hydrophilicity. Additionally, SS is leveraged to amplify the biocompatibility and enhance the encapsulation efficacy of ciprofloxacin (CIP), a critical feature for effectively targeting medical pathogens. Moreover, incorporating SS may modify the microstructure of the PCL matrix, potentially leading to a more heterogeneous distribution. This can result in additional microdomains or an expanded surface area that is more prone to degradation. 

The inclusion of PVA in this experiment serves multiple critical functions. Primarily, PVA enhances the hydrophilicity of the nanofibre, which is essential for promoting tissue integration in wound dressing applications. Additionally, PVA acts as an emulsifier, stabilizing the emulsion and ensuring uniform distribution of components within the nanofibre matrix. Its film-forming capability further contributes to the structural integrity and functional performance of the dressing [47,48]. Moreover, the presence of PVA improves the wetting characteristics of the nanofibre, addressing the challenge of slow degradation that would otherwise occur without PVA. This balance between hydrophilicity and controlled degradation is crucial for the effective use of the nanofibre in wound healing. The role of Tween 20 is to ensure the uniformity of the composite, essential for consistent drug delivery, by exhibiting supplementary emulsifying features [49]. The inclusion of CIP addresses its therapeutic role as an effective antibiotic specifically against medically significant pathogens such as *S. aureus* and *P. aeruginosa*, known for their clinical relevance and resistance profiles. The structural framework provided by PCL affords the nanofibre scaffolds the requisite mechanical properties for potential clinical applications in infection control and tissue engineering.

Polycaprolactone (PCL) was selected as the matrix material in this study due to its outstanding mechanical strength and stability, which are crucial for maintaining the structural integrity of tissue engineering scaffolds over extended periods. While hydrophilic polymers are often favored for their biocompatibility and capacity for effective tissue integration, they typically lack the mechanical robustness that PCL offers. The hydrophobic nature of PCL may seem disadvantageous for biomedical applications requiring hydrophilicity; however, this limitation is offset by its ability to be chemically modified by incorporating hydrophilic segments, thereby enhancing its surface properties without compromising its structural benefits. PCL is inherently hydrophobic, presenting challenges for its direct use in biomedical applications where hydrophilicity is often required for effective tissue integration [50]. The slower degradation rate of PCL compared to hydrophilic polymers is another significant advantage, particularly for applications requiring controlled drug release. This characteristic ensures a prolonged therapeutic effect, which is critical for chronic wound management. Moreover, FDA-approved status of PCL provides an added layer of safety and regulatory compliance, making it a more reliable choice over other materials that might require extensive validation.

On the contrary, SS is hydrophilic, and its divergent nature makes it difficult to form a stable blend. This experimental design aims to address and reconcile these contrasting properties. To do so, CIP is first encapsulated within an SS matrix with the addition of PVA, creating a hydrophilic drug delivery system (Figure S1). This SS-PVA-CIP complex is then slowly introduced, dropwise, into the PCL solution with Tween 20. Subsequent probe sonication ensures the mixture reaches a homogenous state. Tween 20, alongside PVA within the nanofibre formulation, plays a crucial role in uniting the hydrophobic PCL and the hydrophilic SS-PVA-CIP, allowing for the formation of a cohesive, integrated network suitable for medical applications.

Several factors are crucial in the successful fabrication of nanofibres, including the viscosity and conductivity of the emulsion—the flow rate, applied voltage, and distance between the nozzle and the collector [51]. The viscosity of the polymer emulsion is crucial; it dictates whether the output will be fibrous or take the form of droplets—the latter potentially resulting in a thin film rather than a fibrous scaffold. The flow rate and voltage must be carefully optimized to ensure the formation of a stable Taylor cone, which is essential for drawing out the polymer strand toward the collector [52]. An inadequate flow rate might prevent the Taylor cone from forming. Conversely, a higher voltage enhances the electrostatic forces that propel the polymer solution toward the collector, facilitating the fiber elongation and deposition process [53]. Meanwhile, the nozzle-to-collector distance is a determinant of the deposition area of the nanofibre, affecting the fibre’s drying time and the final morphology of the scaffold. Adjusting this distance is critical for ensuring that the fibres do not land on the collector too wet or too dry, thus promoting a uniform scaffold structure [54].

The PCL concentration was set at 10% (*w*/*v*) in ethyl acetate, a choice informed by the literature indicating that this parameter will yield the formation of nanofibres with high mechanical properties, notably high ultimate tensile strength [42]. To explore the influence of SS on the scaffold on cytocompatibility, biodegradability, and antibacterial efficacy, we examined two SS concentrations: 1% and 2%. The selection was guided by research suggesting a minimum of 1% SS to prevent premature gelation, enhancing the viscosity of the electrospinning solution to fall within the ideal range for fiber formation (0.1–20 Pa·s) [55]. This adjustment aimed to produce smoother, more uniform nanofibres. A 4:1 (*v*/*v*) ratio of PCL/SS in the emulsions was employed across all experiments, proving effective in improving cell adhesion, proliferation, and the overall morphology of the nanofibres.

**Figure 1:**
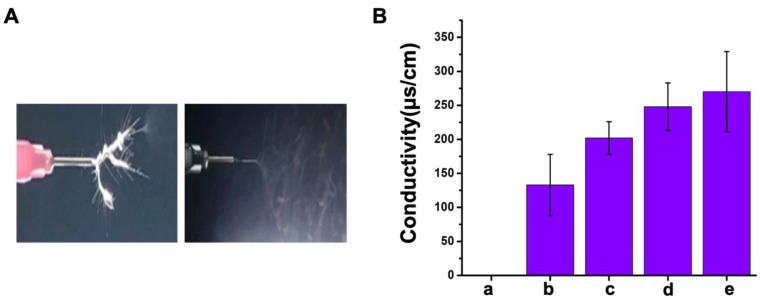
(**A**) Effect of fast-evaporating solvents on conical-shaped polymeric droplet during electrospinning and stable Taylor cone formation with control of T and RH in the chamber. (**B**) Conductivity of (a) 10% PCL, (b) 1% PCL/SS-PVA-CIP (3.0 μg/g), (c) PCL/1% SS-PVA-CIP (7.0 μg/g) (d) PCL/2% SS-PVA-CIP (3.0 μg/g), and (e) PCL/2% SS-PVA-CIP (7.0 μg/g). Data illustrates mean ± SD, n = 3.

Optimizing the electrospinning parameters—such as the distance between the needle and collector, applied voltage, and solution flow rate—was crucial for fabricating the PCL/SS nanocomposite. The conductivity of the polymeric solution, a critical factor affecting fiber formation, was adapted from previous studies [56]. The inherently low conductivity of PCL dissolved in ethyl acetate (<0.039 μS/cm for 10% PCL) (Figure 1B) was compensated by the rapid evaporation rate of the solvent, especially compared to the SS-PVA-CIP solution in a dry chamber setting (Figure 1A). System parameters were finely tuned to counteract the challenges posed by the low boiling point of ethyl acetate and the high viscosity of the SS solution, attributed to the addition of Tween 20 as a stabilizing surfactant [49]. The voltage and flow rate adjustments aimed to maintain steady jet formation from the needle by balancing the exit and inflow rate of the polymer solution, with the flow rates set between 0.8 mL/h and 1 mL/h [57]. The voltage was increased from 20 kV to 23 kV, and the flow rate was reduced to the range 0.3 mL/h to 0.5 mL/h to enhance electrostatic forces, minimize fiber cooling time, and ensure the formation of a continuous jet by reducing the viscosity of the emulsion. The morphology and structural integrity of electrospun fibres are significantly influenced by the distance between the nozzle and the collector, which is shown in Figure S2. Table 2 tabulates the summary of the optimized parameters of this experiment.

### 3.2. Fiber Morphology

**Figure 2:**
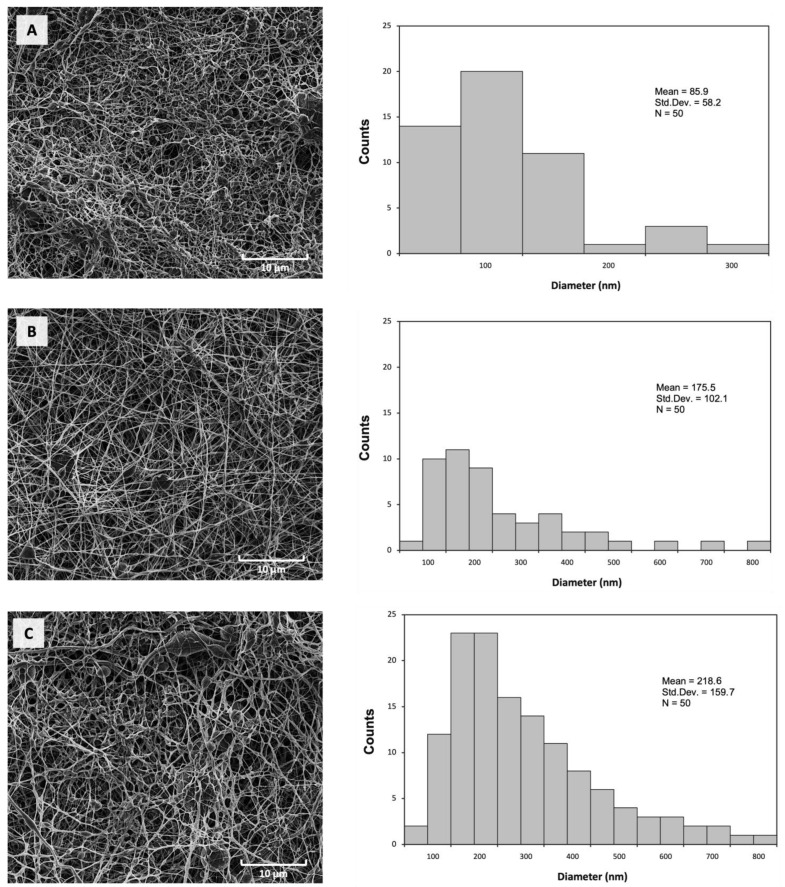
SEM images (**left**) and diameter distributions (**right**) showing morphological features of electrospun PCL scaffolds. The scaffolds were obtained from 10% *w*/*v* pure PCL in ethyl acetate, with 2% *w*/*v* SS, 0.5% *w*/*v* PVA, and (**A**) 1.5 μg/g, (**B**) 3.0 μg/g and (**C**) 7.0 μg/g of CIP. Fiber diameter distribution of (**A**–**C**) scaffolds with mean ± SD for 50 nanofibres.

The SEM images depicted in Figure 2 demonstrate a direct correlation between the viscosity of the fiber solution and its resultant diameter, providing a compelling visualization of the impact of emulsion properties on fiber morphology. Specifically, Figure 2B,C illustrate that an increase in antibiotic concentration correlates with elevated emulsion viscosity, leading to an increase in fiber diameter. This observation emphasizes the direct influence of the SS-PVA-CIP concentration on the dimensional attributes of the fibres, where the increased surface charge associated with higher SS-PVA-CIP concentrations enhances the solution’s conductivity. Consequently, this increases the electrostatic repulsion forces under the influence of the applied voltage, facilitating further elongation of the nanofibre, and leading to the production of smoother and thicker fibres.

**Figure 3:**
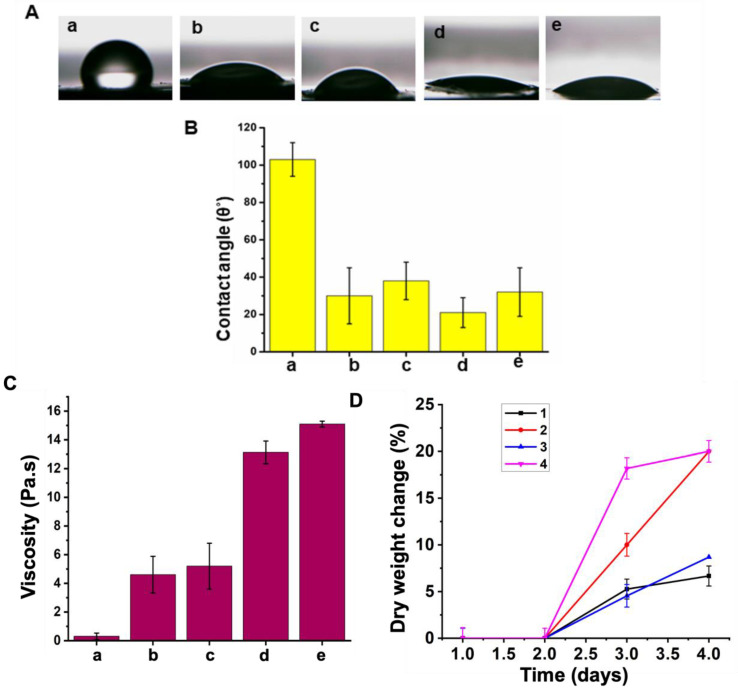
(**A**) Contact angle drop images and (**B**) measured contact angle values, (**C**) viscosity, and (**D**) conductivity of (**a**) 10% PCL, (**b**) 1% PCL/SS-PVA-CIP (3.0 μg/g), (**c**) PCL/1% SS-PVA-CIP (7.0 μg/g) (**d**) PCL/2% SS-PVA-CIP (3.0 μg/g), and (**e**) PCL/2% SS-PVA-CIP (3.0 μg/g). Data illustrate mean ± SD, n = 3. (**D**) The graph showing the change in dry weight of electrospun scaffolds due to the degradation: (1) PCL/1% SS-PVA-CIP (3.0 μg/g), (2) PCL/1% SS-PVA-CIP (7.0 μg/g), (3) PCL/2% SS-PVA-CIP (3.0 μg/g) and (4) PCL/2% SS-PVA-CIP (7.0 μg/g) over 1 week in PBS (n = 3).

Moreover, the SEM analyses indicate that the necessary adjustments in voltage and flow rate to mitigate the effects of surface tension are modulated downwards in response to the increased viscosity imposed by higher concentrations of the polymeric emulsion. This phenomenon is exemplified in SEM images corresponding to viscosities of 10.5 Pa·s (Figure 2A), 13.13 Pa·s (Figure 2B) and 125 Pa·s (Figure 2C). As the viscosity increases, a resulting reduction in the applied voltage (from 20–22 kV to 12–18 kV) and flow rates (from 0.5–1 mL/h to 0.3–0.5 mL/h) is observed (Figure 3C). This trend is evident across the spectrum of fiber diameters, with a marked increase in average fiber diameter from 85.94 ± 58.21 nm, 175.48 ± 102.1 nm and 218.58 ± 159.67 nm for each concentration of CIP, respectively (1.5 μg/g, 3.0 μg/g and 7.0 μg/g), as showcased in Figure 2. This relationship between fiber diameter and polymer emulsion viscosity aligns with the findings of previous research. For instance, an earlier study documented a significant increase in the diameter of electrospun polyacrylonitrile (PAN) precursor fibres from 588 nm to 690 nm as the polymer concentration escalated from 8 wt% to 12 wt% [58]. Another investigation highlighted the role of increasing SS content in promoting fiber solidification through the non-solvent effects of water, facilitating the formation of fibres with increased diameters [43]. The results presented herein are in concordance with these and other studies, reinforcing the established understanding of the influence of solution viscosity on fiber morphology [59,60].

Nevertheless, in addition to the presence of thinner fibres, the occurrence of bead formation was more prominent when the viscosity surpassed a crucial threshold, as evidenced in Figure 2C. These beads are anticipated to act as reservoirs for encapsulating drugs, improving the stability of the membrane by preventing fiber aggregation, increasing the surface area and enhancing the performance of the scaffolds. Bead formation can be controlled by careful optimization of electrospinning parameters, as discussed above. Overall, the interconnected pore structure, nanograde nature and bead formation can be garnered for tissue regeneration as nanoscale fibres can mimic the fibrous architecture of native tissues.

### 3.3. Hydrophilicity

The assessment of surface wettability, as quantified by the contact angle measurement, is a critical determinant of the biocompatibility of nanocomposites, crucially influencing cell adhesion, protein adsorption, and overall material integration within biological environments [61]. This parameter is determined by the chemical composition, surface charge, and microstructure of the scaffold, each contributing to its interaction with the biological environment [36,62].

Surface wettability is typically categorized into three distinct ranges based on the contact angle: hydrophilic (0–30°), moderately hydrophilic (30–90°), and hydrophobic (>90°). It is well-documented in the literature that surfaces with hydrophilic characteristics are more conducive to cell adhesion, proliferation, and differentiation [63,64,65]. Specifically, a study highlighted a decrease in osteoblast adhesion and proliferation on surfaces as they transitioned from hydrophilic to hydrophobic (0 to 106°) [66]. Additionally, optimal fibroblast adhesion has been associated with contact angles within the range of 60° to 80° [64,67]. Given that both extracellular matrix syntheses require hydrophilic surfaces, this underscores the importance of tailored surface properties for enhanced cellular activities.

In this investigation, we have demonstrated that the incorporation of SS significantly enhances the hydrophilic properties of the scaffolds, with increased SS concentrations correlating with improved surface wettability (Figure 3A). Our results revealed that 10% *w*/*v* PCL scaffolds presented a contact angle of 103 ± 9°, indicative of inherent hydrophobicity, which was expected. In contrast, scaffolds formulated with 1% SS-PVA-CIP (3.0 μg/g of PCL and 2% SS-PVA-CIP (3.0 μg/g)/PCL displayed contact angles of 30 ± 15° and 21 ± 8°, respectively, signifying a shift towards more hydrophilic behavior (Figure 3B). The enhanced wettability can be attributed to the hydroxyl-rich side chains of SS molecules, which interact with the PCL surface to increase hydrophilicity [56]. Furthermore, the inherent hydrophilicity of SS is derived from its composition, including aspartic and serine acids [68].

An increase in the concentration of CIP was observed to inversely affect the water affinity, with the contact angle rising from 21° to 32° as the antibiotic concentration increased from 3.0 μg/g to 7.0 μg/g (Figure 3B). This phenomenon is likely due to the inherent hydrophobic nature and limited solubility of CIP, aligning with findings from previous studies [69]. Despite this, the electrospun PCL/SS-PVA-CIP nanofibrous scaffolds are characterized as hydrophilic nanocomposites, facilitating cell adhesion and proliferation.

### 3.4. Mass Loss

We assessed the initial degradation behavior of electrospun scaffolds under physiological conditions. This was achieved by monitoring changes in the dry weight of the scaffolds to quantify their stability and degradation kinetics when exposed to an environment mimicking physiological conditions.

As shown in Figure 3D, in the first 24 h, no significant change in dry weight was observed for electrospun scaffolds containing either 1% or 2% SS, irrespective of the antibiotic concentration. However, as the exposure period extended to 3 days, a noticeable loss of mass pattern emerged, particularly in scaffolds with a higher concentration of 2% SS, which exhibited a greater percentage of weight loss compared to their 1% SS counterparts. This trend continued over 7 days, where scaffolds with 1% SS maintained their weight across both antibiotic concentrations, whereas those with 2% SS experienced further degradation.

**Scheme 2:**
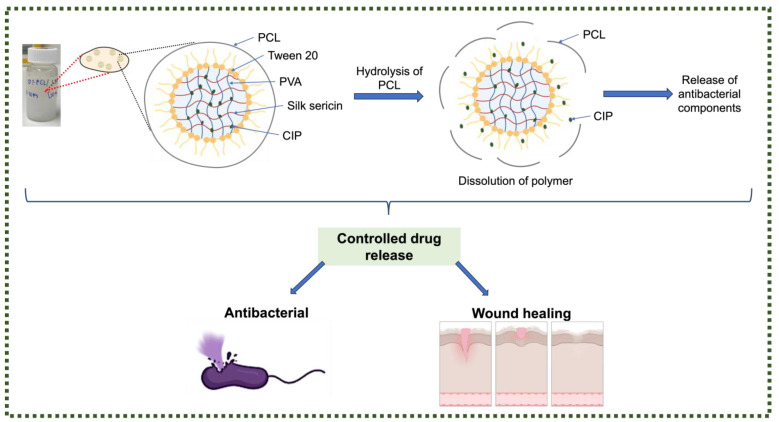
The process and mechanism of slow drug release of the nanofibre scaffolds and its applications.

The accelerated degradation rate in scaffolds with a higher SS concentration suggests that the increased ratio of SS to PCL enhances the scaffold’s biodegradability. Scheme 2 illustrates the controlled drug release mechanism from the nanofibre scaffolds, designed to optimize therapeutic effects. The scaffold composition, primarily PCL, is key due to the properties of PCL as a biodegradable polymer that effectively incorporates bioactive components. Given that PCL alone demonstrates limited degradation properties, the integration of natural polymers like SS is shown to significantly augment its biodegradability [43]. As shown in Figure 3A,B, increasing the concentration of SS and incorporating PVA enhances the hydrophilicity of the scaffolds. This increased hydrophilicity is critical, as it ensures that upon immersion in an aqueous environment, the scaffolds facilitate a gradual release of encapsulated antibiotics. This mechanism mitigates the burst release typically observed with less hydrophilic materials, promoting a more controlled drug release. The scaffold’s slow water absorption permits a steady diffusion of CIP from regions of higher concentration within the delivery system to lower concentrations in surrounding tissues or fluids. This diffusion, driven by the drug concentration gradient, is the primary mode of release. Moreover, as the scaffold degrades or is absorbed by the body, this process further modulates the CIP release rate, emphasizing the importance of synchronizing the degradation timeline with drug release to maintain therapeutic levels throughout the treatment period.

Therefore, the profiles of both the 1% SS-PVA-CIP/PCL and 2% SS-PVA-CIP/PCL scaffolds affirm their stability in PBS solutions. This stability, coupled with their demonstrated suitability for cellular compatibility and proliferation, as verified by the MTT assay, underscores their potential for in vivo applications.

### 3.5. Antibacterial Activity

The antibacterial activity of PCL and drug-loaded PCL-based composite scaffolds was evaluated using a ZOI assay. *P. aeruginosa* and *S. aureus* were the model bacteria used for this study as they were the principal causative agents of bacterial wound infections.

**Figure 4:**
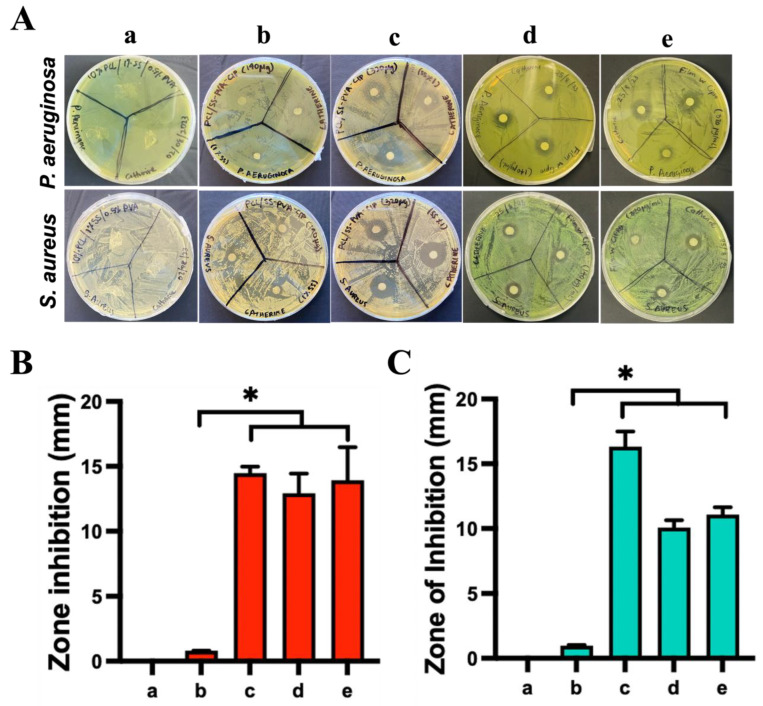
Photographic images of (**A**) ZOI of (**a**) PCL (negative control), (**b**) PCL/1% SS-PVA-CIP (3.0 μg/g), (**c**) PCL/1% SS-PVA-CIP (7.0 μg/g), (**d**) PCL/2% SS-PVA-CIP (3.0 μg/g), and (**e**) PCL/2% SS-PVA-CIP (7.0 μg/g) exposed to *P. aeruginosa* and *S. aureus*. (**B**,**C**) Bar graphs representing ZOI of (**a**) PCL (negative control), (**b**) PCL/1% SS-PVA-CIP (3.0 μg/g), (**c**) PCL/1% SS-PVA-CIP (7.0 μg/g), (**d**) PCL/2% SS-PVA-CIP (3.0 μg/g), and (**e**) PCL/2% SS-PVA-CIP (7.0 μg/g) exposed to *P. aeruginosa* and *S. aureus*, respectively. Data were analyzed using one-way ANOVA and Tukey analysis; data represent mean ± SD, n = 3, * *p* ≤ 0.05.

Optimal CIP concentrations for targeting *P. aeruginosa* and *S. aureus* were identified as 3.0 μg/g and 7.0 μg/g, respectively, through MIC studies (Figure S3). These concentrations were notably higher than conventional dosage levels as the nanofibre intended to gradually release the antibiotic for a long duration. The quantitative analysis in Figure 4 showed that antibacterial action is concentration dependent. The composite scaffolds with the highest loading of CIP, 7.0 μg/g, showed better antibacterial activity on both *P. aeruginosa* and *S. aureus*. It is also evident that bare PCL did not show any antibacterial effect on either strain due to the absence of CIP, which was expected [70]. Comparing composite scaffolds containing 1% and 2% SS, samples containing 2% SS showed a better antibacterial effect, demonstrating its reinforcing effect. Silk sericin can act as a porogen, creating pores within the PCL matrix. Higher concentrations of sericin can lead to more significant porosity, allowing for easier diffusion of the antibiotic molecules out of the emulsion.

The electrospun PCL/SS-PVA-CIP nanofibrous scaffolds exhibit exceptional stability and drug delivery capabilities, primarily due to the hydrophilic nature and unique configuration of SS. These scaffolds are particularly suitable for drugs with low solubility, like CIP, as they stabilize the drugs through molecular interactions, including electrostatic interactions and hydrogen bonding. Previous research has explored the role of SS in enhancing the stability of drugs within binary solid dispersions through these intermolecular forces, highlighting the multifunctional application of these nanofibres in antimicrobial therapy [71].

### 3.6. Cytocompatibility

Biocompatible scaffolds are necessary for tissue engineering to provide essential support for cellular growth and regeneration. Among the materials used, SS stands out for its capacity to enhance cell proliferation, thus contributing significantly to the development of functional tissue engineering scaffolds [72,73]. The nanofibres prepared in this study leverage the biofunctional advantages of SS, combined with a synthetic polymer blend, to preserve scaffold integrity. This strategic composition prevents premature fiber degradation, thereby ensuring the scaffold’s efficacy in its designated applications.

**Figure 5:**
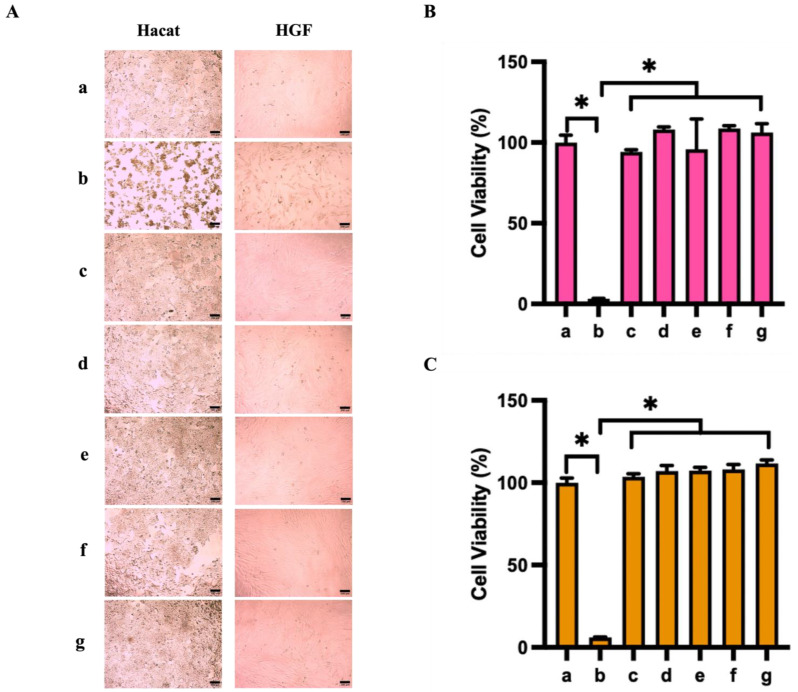
(**A**) The proliferation of HaCaT and HGF cells under multiple treatments and (**B**,**C**) MTT assay results of (**a**) TCP (vehicle control), (**b**) 6.4 μg/mL CIP (positive control), (**c**) PCL (negative control), (**d**) PCL/1% SS-PVA-CIP (3.0 μg/g), (**e**) PCL/2% SS-PVA-CIP (3.0 μg/g), (**f**) PCL/1% SS-PVA-CIP (7.0 μg/g) and (**g**) PCL/2% SS-PVA-CIP (7.0 μg/g). Data were analysed using one-way ANOVA and Tukey analysis, n = 6; mean ± SD; * *p* ≤ 0.05.

To assess the potential cytotoxic effects of the scaffolds containing SS, we conducted a comparative analysis of the cytotoxicity exhibited by electrospun scaffolds with 1% and 2% SS towards HaCaT and HGF cells. These cells were treated with scaffolds of CIP at concentrations of 3.0 μg/g and 7.0 μg/g. Utilizing the MTT assay, with 10% PCL serving as a negative control, 6.4 μg/mL CIP as a positive control, and a tissue culture plate (TCP) as a standard, we evaluated cellular responses. Figure 5 showcases the cellular morphology of both cell lines on the scaffolds after an overnight incubation, revealing that cells treated with PCL/SS-PVA-CIP composite scaffolds did not exhibit adverse effects. On the contrary, there was a notable increase in growth rates, exceeding 100% for both concentrations of CIP tested. However, it was observed that both cell lines displayed the lowest viability at the highest CIP concentration (7.0 μg/g) directly applied, an effect not mirrored in cells treated with the same concentration of CIP when delivered via the SS-containing nanofibres [74,75]. This variation underscores the protective role of SS against antibiotic-induced cytotoxicity. Notably, cells treated with scaffolds containing 2% SS exhibited improved morphology over those treated with scaffolds containing 1% SS, indicating that higher concentrations of SS afford greater cytoprotective benefits [76].

The PCL scaffolds demonstrated no cytotoxic effects on either cell line, with HaCaT and HGF proliferation rates exceeding 80% and 100%, respectively, as illustrated in Figure 5.

This result signifies that there was no significant variance in cell proliferation between the control groups and the PCL scaffold, confirming the biocompatibility of the scaffold. The minimal proliferation observed in the presence of the positive control (CIP alone) highlights the potential cytotoxic risks associated with the uncontrolled release of CIP. The gradual release of CIP from the nanofibrous scaffolds enabled effective proliferation rates in HaCaT and HGF cells, even at elevated CIP concentrations.

Furthermore, an increase in SS concentration led to a marked improvement in cell proliferation, with HaCaT cells showing a 9% proliferation increase and HGF cells a 12% increase at the respective CIP concentration compared to TCP. This enhancement in cell proliferation attributed to the SS-containing scaffolds emphasizes the beneficial impact of SS on cellular attachment and growth, matching findings from similar studies that have demonstrated the efficacy of higher SS levels in promoting adhesion and proliferation of human primary skin fibroblast cells [76].

## 4. Conclusions

The primary aim of this study was to investigate the optimization and influence of key parameters in the development of ciprofloxacin-impregnated nanocomposites using electrospinning techniques. Our findings demonstrate that the integration of SS significantly enhances the hydrophilicity and, consequently, the surface wettability of the scaffolds. However, it was observed that an increase in antibiotic concentration inversely affected the hydrophilicity. The CIP nanocomposites, in synergy with SS, displayed potent antibacterial activity against *P. aeruginosa* and *S. aureus*, identifying 3.0 μg/g or 7.0 μg/g as the most efficacious CIP concentrations—substantially below conventional dosage thresholds. Additionally, escalating the antibiotic concentration within a specified range intensified the antibacterial efficacy. The biocompatibility of these nanofibres with HaCaT and HGF cell lines was exceptional, showcasing significant cellular proliferation even at the higher antibiotic dose of 7.0 μg/g. This study not only highlights the potential of electrospun nanofibres for treating wound infections but also lays a crucial groundwork for further enhancing the functionality and therapeutic effectiveness of the material. The compelling outcomes related to drug release, biodegradation, and cell compatibility present these nanofibrous scaffolds as promising therapeutic avenue foundations. This research provides an important theoretical framework for improving material performance and treatment effects in electrospun nanocomposites.

## Figures and Tables

**Table 1:** The final concentration of ciprofloxacin in the electrospun nanofibre mats.

Sample	Initial Concentration (μg/mL)	Final Concentration (μg/g)
1	70	1.5
2	140	3.0
3	320	7.0

**Table 2:** Summary of the most optimium electrospinning parameters used to generate nanofibres.

Viscosity	Conductivity	Environmental Condition	Distance
10–15 Pa·s	>100 μS/cm	T: 23 °C	12.5 cm

## Data Availability

The data that support the findings of this study are available from the corresponding author upon reasonable request.

## Supplementary Materials

The following supporting information can be downloaded at https://www.mdpi.com/article/10.3390/nano14171429/s1: References [77,78,79,80,81,82,83,84] are cited in the supplementary materials.
