# Supplementary material for: Antibacterial Electrospun Membrane with Hierarchical Bead-on-String Structured Fibres for Wound Infections
Source: Nanomaterials (Basel). 2024 Aug 31;14(17):1429. doi: 10.3390/nano14171429 (PMC11397722; doi:10.3390/nano14171429)
Supplement: Supplementary file 1 [file nanomaterials-14-01429-s001.zip › nanomaterials-3167969-supplementary.pdf]

Antibacterial core-shell electrospun membrane with the hierarchical bead on string structured fibres for wound infections

Yu Xuan Fong[1], Catherine Pakrath[1], Fathima Shana Pattar Kadavan[1], Tien Thanh Nguyen[1], Trong Quan Luu[1], Borislav Stoilov[1], Richard Bright[1], Manh Tuong Nguyen[1], Neethu Ninan[1], Youhong Tang[2], Krasimir Vasilev[1*], Vi Khanh Truong[1],*

1 College of Medicine and Public Health, Flinders University, Bedford Park, Adelaide, SA 5042, Australia; fong0107@flinders.edu.au (Y.X.F.); cpakrath@gmail.com (C.P.); patt0238@flinders.edu.au (F.S.P.K.); nguy1184@flinders.edu.au (T.T.N.); luu0044@flinders.edu.au (Q.T.L.); borislav.stoilov@flinders.edu.au (B.S.); richard.bright@flinders.edu.au (R.B); ManhTuong.Nguyen@flinders.edu.au (M.T.N.); neethun.ninan@gmail.com (N.N.)

2 Institute for NanoScale Science and Technology, Medical Device Research Institute, College of Science and Engineering, Flinders University, Bedford Park, Adelaide, SA 5042, Australia; youhong.tang@flinders.edu.au (Y.T.)

* Correspondence: krasimir.vasilev@flinders.edu.au (K.V.); (vikhanh.truong@flinders.edu.au (V.K.T.)

Supplementary Figures

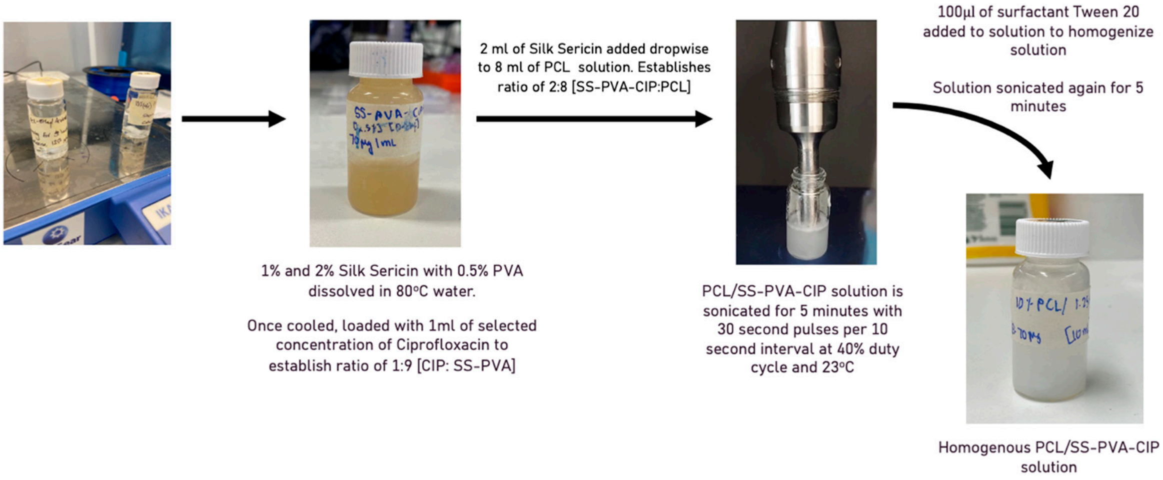

Figure S1. Schematic diagram of the preparation process of the polymer solution containing polycaprolactone, silk sericin, ciprofloxacin, poly(vinyl alcohol), and Tween 20.

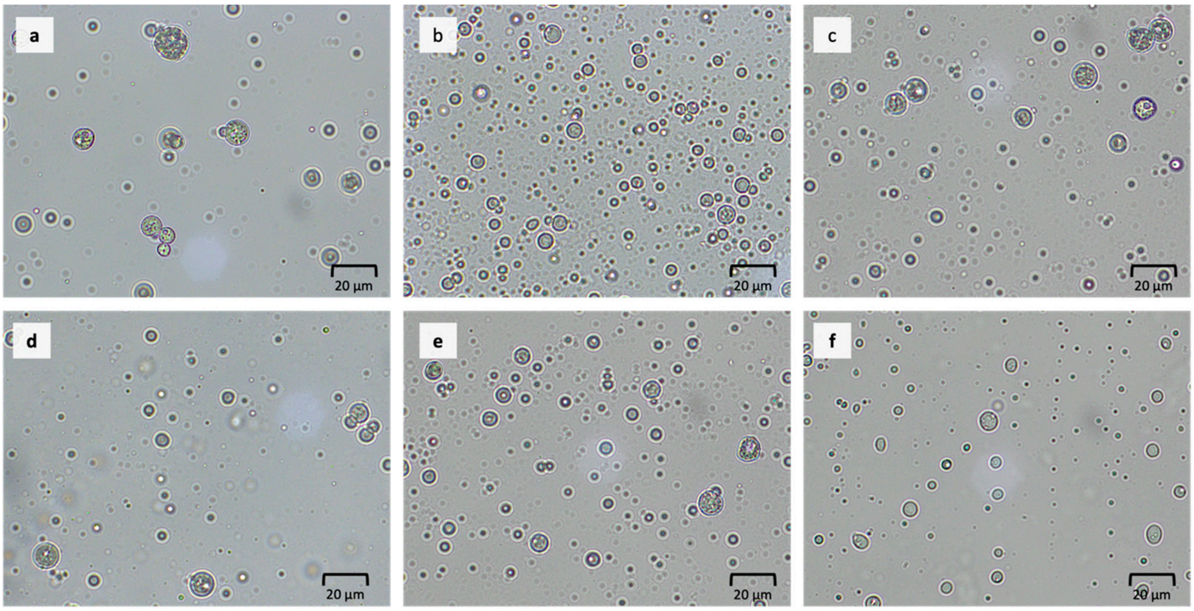

21

Figure S2. Optical microscopy images of the oil-in-water emulsions of 1% SS-PVA **a)** 1.5 µg/g, **b)** 3.0 µg/g, **c)** 7.0 µg/g and 2% SS-PVA **d)** 1.5 µg/g, **e)** 3.0 µg/g and **f)** 7.0 µg/g.

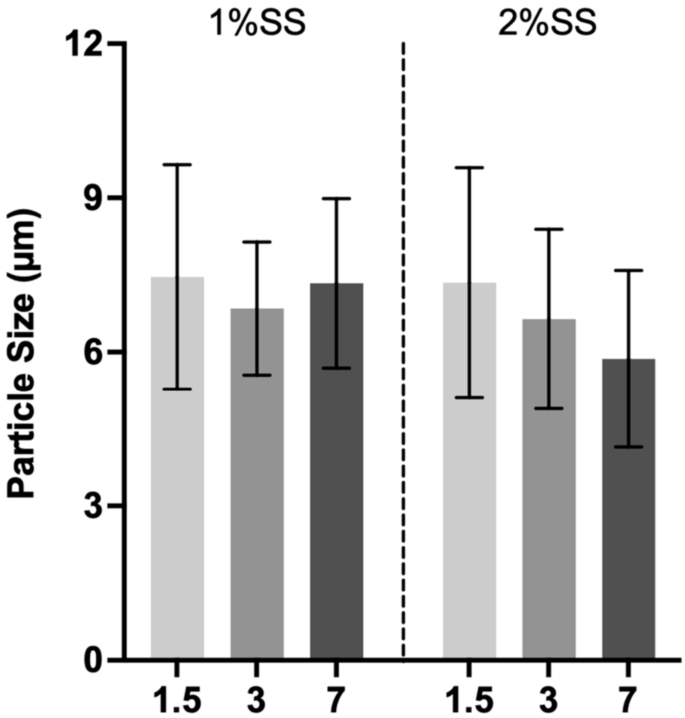

Figure S3. The particle size distribution of oil-in-water of the electrospinning emulsion of different concentrations of silk sericin and ciprofloxacin.

The average particle size distribution was measured at 7.028 ± 1.864 μm, with no significant variation observed across samples. Importantly, the particle size was not influenced by the concentration of silk sericin or ciprofloxacin (CIP).

Table S1. The sample naming for Figure 1, Figure 3, Figure 4 and Figure 5.

Sample	Component
**a**	10% PCL
**b**	PCL/1% SS-PVA-CIP (3.0 μg/g)
**c**	PCL/1% SS-PVA-CIP (7.0 μg/g)
**d**	PCL/2% SS-PVA-CIP (3.0 μg/g)
**e**	PCL/2% SS-PVA-CIP (7.0 μg/g)

Effect of the distance between the nozzle and the collector

The structural nature of electrospun fibers is significantly influenced by the distance between the nozzle and the collector [1, 2]. This parameter critically affects factors including the deposition time, evaporation rate, and the interval of whipping or instability, of all which play a role in determining the final characteristics of the nanofibers [3]. In this investigation, the effect of varying spinning distances on the structure and dimensional properties of PCL/SS-PVA-CIP nanofibers was evaluated. The study utilized SS solutions at concentrations of 1 wt% and 2 wt%, with spinning distances spanning from 10 to 15 cm, reflecting common ranges reported in the literature [4-7].

SEM provided visual documentation of the nanofibers fabricated under these conditions, revealing insights into the influence of spinning distance on fiber morphology. Consistent with existing literature, an increase in the distance between the nozzle and the collector typically leads to a reduction in fiber diameter [8]. In this study, an increase in distance resulted in a notable decrease in the diameter of fibers from the 1% SS solution (viscosity of 4.6 Pa·s), shrinking from 190 nm to 46 nm, thereby achieving enhanced uniformity. This reduction in diameter is likely due to the complete evaporation of the solvent, facilitating greater stretching and thinning of the fibers. Conversely, employing a 2% SS solution with a higher viscosity of 13.13 Pa·s led to a reduction in fiber diameter within the range of 275 nm to 95 nm. This behaviour can be ascribed to the pronounced shear thinning effect, exacerbated by the viscoelastic properties of the more concentrated solution. Optimal fiber homogeneity and size for both SS concentrations were achieved at a spinning distance of 12.5 cm. This optimal distance allows for adequate fiber cooling and solidification while maintaining a suitable deposition time that mitigates excessive solvent evaporation, which could occur at distances exceeding the critical threshold.

Therefore, the structure of the membrane is found to depend on both the viscosity and distance of the polymer solution. As the viscosity and distance grow, the porosity of the membrane gradually increases. Along with this trend, structures that look like beads are becoming less common and structures that look like spindles are becoming firmer.

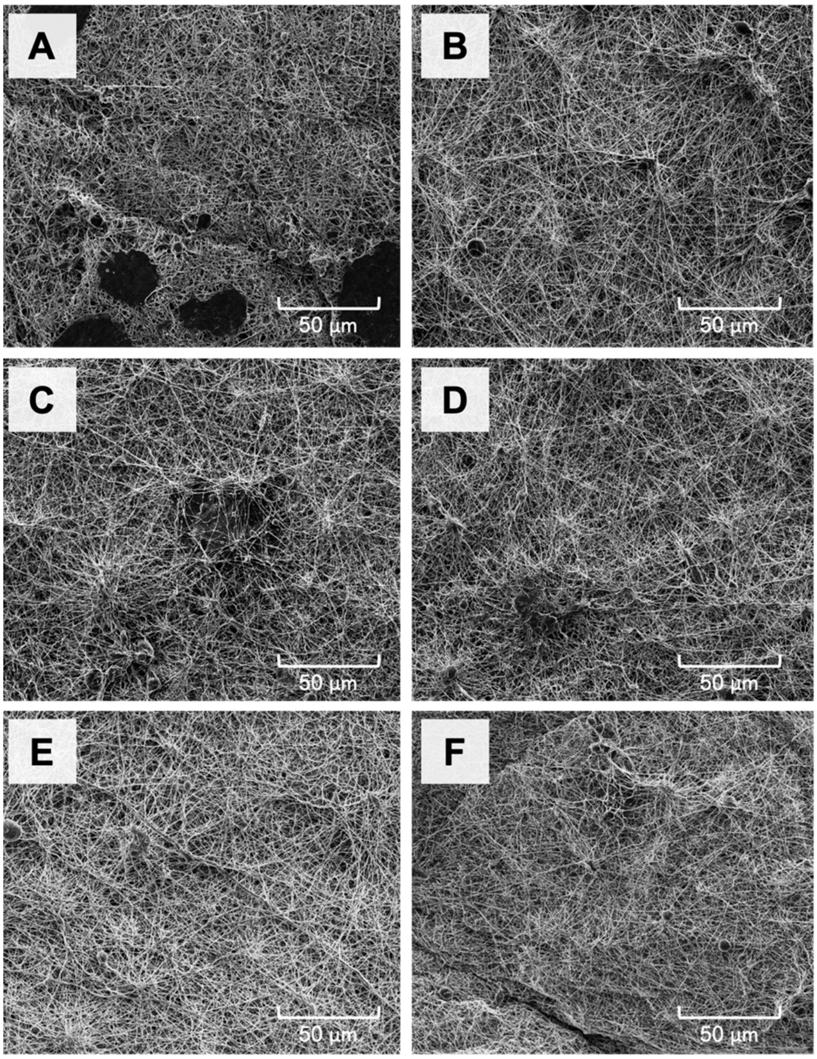

Figure S4. SEM images show the structural details of electrospun mats, including insets in the bottom right at a 1-micrometer scale, featuring scaffolds impregnated with Ciprofloxacin (3.0 µg/g) within 1% SS-PVA-CIP/PCL and 2% SS-PVA-CIP/PCL composites. These images correspond to varied spinning distances: for 1% SS composites at (A) 10 cm, (B) 12.5 cm, and (C) 15 cm, and for 2% SS composites at (D) 10 cm, (E) 12.5 cm, and (F) 15 cm.

Table S2. The minimum inhibition concentration (MIC) of CIP on *P. aeruginosa* and *S. aureus*.

Bacterial strains	MIC (µg/mL)
P. aeruginosa	0.25
S. aureus	0.25

Reference:

1. Badmus, M., et al., *Hierarchically electrospun nanofibers and their applications: A review.* Nano Materials Science, 2021. **3**(3): p. 213-232.
2. Nezarati, R.M., M.B. Eifert, and E. Cosgriff-Hernandez, *Effects of humidity and solution viscosity on electrospun fiber morphology.* Tissue Engineering Part C: Methods, 2013. **19**(10): p. 810-819.
3. Hekmati, A.H., et al., *Effect of needle length, electrospinning distance, and solution concentration on morphological properties of polyamide-6 electrospun nanowebs.* Textile Research Journal, 2013. **83**(14): p. 1452-1466.

4. Fatimah, I., T.I. Sari, and D. Anggoro, *Effect of concentration and nozzle-collector distance on the morphology of nanofibers.* Key Engineering Materials, 2020. **860**: p. 315-319.
5. Chen, M., et al., *Electrospinning of calixarene-functionalized polyacrylonitrile nanofiber membranes and application as an adsorbent and catalyst support.* Langmuir, 2013. **29**(38): p. 11858-11867.
6. Park, J.-Y. and I.-H. Lee, *Controlled release of ketoprofen from electrospun porous polylactic acid (PLA) nanofibers.* Journal of Polymer Research, 2011. **18**: p. 1287-1291.
7. Wen, P., et al., *Fabrication of electrospun polylactic acid nanofilm incorporating cinnamon essential oil/β-cyclodextrin inclusion complex for antimicrobial packaging.* Food chemistry, 2016. **196**: p. 996-1004.
8. Al-Hazeem, N.Z., *Effect of the Distance between the Needle Tip and the Collector on Nanofibers Morphology.* Nanomed. Nanotechnol, 2020. **5**: p. 1-5.
